# Resistance, remission, and qualitative differences in HIV chemotherapy.

**DOI:** 10.3201/eid0303.970303

**Published:** 1997

**Authors:** D E Kirschner, G F Webb

**Affiliations:** Department of Microbiology and Immunology, University of Michigan Medical School, Ann Arbor, MI 48109-0620, USA. kirschne@umich.edu

## Abstract

To understand the role of qualitative differences in multidrug chemotherapy for human immunodeficiency virus (HIV) infection in virus remission and drug resistance, we designed a mathematical system that models HIV multidrug chemotherapy including uninfected CD4+ T cells, infected CD4+ T cells, and virus populations. The model, which includes the latent and progressive stages of the disease and introduces chemotherapy, is a system of differential equations describing the interaction of two distinct classes of HIV (drug-sensitive [wild type] and drug-resistant [mutant]) with lymphocytes in the peripheral blood; the external lymphoid system contributes to the viral load. The simulations indicate that to preclude resistance, antiviral drugs must be strong enough and act fast enough to drive the viral population below a threshold level. The threshold depends upon the capacity of the virus to mutate to strains resistant to the drugs. Above the threshold, mutant strains rapidly replace wild-type strains. Below the threshold, resistant strains do not become established, and remission occurs. An important distinction between resistance and remission is the reduction of viral production in the external lymphoid system. Also the virus population rapidly rebounds when treatment is stopped even after extended periods of remission.


### Resistance, Remission, and Qualitative Differences in HIV Chemotherapy

**Denise E. Kirschner* and G.F. Webb†**
*University of Michigan Medical School, Ann Arbor, Michigan, USA; and
†Vanderbilt University, Nashville, Tennessee, USA

To understand the role of qualitative differences in multidrug chemotherapy for human immunodeficiency virus (HIV) infection in virus remission and drug resistance, we designed a mathematical system that models HIV multidrug chemotherapy including uninfected CD4+ T cells, infected CD4+ T cells, and virus populations. The model, which includes the latent and progressive stages of the disease and introduces chemotherapy, is a system of differential equations describing the interaction of two distinct classes of HIV (drug-sensitive [wild type] and drug-resistant [mutant]) with lymphocytes in the peripheral blood; the external lymphoid system contributes to the viral load. The simulations indicate that to preclude resistance, antiviral drugs must be strong enough and act fast enough to drive the viral population below a threshold level. The threshold depends upon the capacity of the virus to mutate to strains resistant to the drugs. Above the threshold, mutant strains rapidly replace wild-type strains. Below the threshold, resistant strains do not become established, and remission occurs. An important distinction between resistance and remission is the reduction of viral production in the external lymphoid system. Also the virus population rapidly rebounds when treatment is stopped even after extended periods of remission.

Mathematical models provide a means to understand the human immunodeficiency virus (HIV)-infected immune system as a dynamic process. Models formulated as differential equations for the dynamic interactions of CD4+ lymphocytes and virus populations are useful in identifying essential characteristics of HIV pathogenesis and chemotherapy. Recent clinical studies have produced new insight into the dynamics of these virus populations during HIV infection (1-3). Turnover rates and lifespans of infected CD4+ T cells and virus have been identified by measuring their rates of change in patients undergoing strong antiviral mono-therapy. The determination of these rates showed that large numbers of CD4+ T cells and virus are gained and lost each day throughout the course of HIV infection (4,5); these findings have profoundly influenced strategies for therapy (6). It is now recognized that chemotherapeutic agents must strongly suppress viral production before rapidly appearing viral mutants evolve to drug resistance. Recent clinical trials have accomplished this goal by using combined drug therapy. Ongoing trials with combinations of drugs have shown sharp declines, in some patients, of viral counts to nondetectable levels within several weeks of treatment; these levels were sustained for 1 year or more (5,7,8). At the same time, CD4+ T-cell counts have risen markedly before gradually leveling off (5,7,8). This apparent remission of HIV infection offers hope for the chronic control or even eradication of HIV (6). The issue of stopping treatment after such extended periods of remission, however, is yet to be resolved (8).

Address for correspondence: Denise E. Kirschner, Department of Microbiology and Immunology, University of Michigan Medical School, 6730 Medical Science Building II, Ann Arbor, MI 48109-0620 USA; fax: 313-764-3562; e-mail: kirschne@umich.edu.

### The Model

Our model of treatment distinguishes qualitatively two treatment outcomes indicated by clinical trials. The first is resistance. Examples of resistance for three-drug combined therapy are reported for completed clinical trials by Collier et al. (9). In these trials, there was on average an increase of CD4+ T-cell counts by approximately 30% (peaking at approximately 8 weeks and returning to baseline at approximately 40 weeks) and a decrease of plasma virus by approximately

70% (peaking at approximately 4 weeks and recovering to half baseline at approximately 40 weeks). The second treatment outcome is remission. Examples of remission are indicated in preliminary reports of ongoing clinical trials (5,7,8,10). In these trials, 1) plasma virus decreased sharply to nondetectable levels in 2 to 4 weeks, and these levels were sustained for periods of 1 year or more, and 2) CD4+ T-cell counts increased steadily by $100/mm^3$ or more before gradually leveling off to below normal levels (this below normal recovery is believed to be due to an impaired production of new CD4+ T cells from the thymus and other sources [11,12]; this assumption is incorporated into the model).

The model consists of differential equations for the variables T(t) (the CD4+ T-cell population uninfected by virus at time t), $T_s(t)$ (the CD4+ T-cell population infected by drug-sensitive virus at time t), $T_r(t)$ (the CD4+ T-cell population infected by drug-resistant virus at time t), $V_s(t)$ (the drug-sensitive virus population at time t), and $V_r(t)$ (the drug-resistant virus population at time t). All these virus populations reside in the circulating blood, in which the values of uninfected CD4+ T cells and virus can be clinically measured. The assumptions of the model and its equations are given in the Appendix.

The model incorporates recent clinically determined dynamic information about the HIV-infected immune system. The essential elements are as follows. After an initial period of acute viremia in the first few weeks after seroconversion, CD4+ T-cell counts decline gradually from approximately 600 to $800/mm^3$ to $0/mm^3$ over approximately 10 years (11) (normal CD4+ T-cell counts are 800 to $1,200/mm^3$). The decline of CD4+ T cells is more rapid early in the infection (13). Infected CD4+ T cells constitute 4% or less of the CD4+ T-cell population (14). The half-life of an infected CD4+ T cell is approximately 2 days (1-3,6). After the initial viremia, plasma virus increases from below $50/mm^3$ to $1,000/mm^3$ or more during the variable course of infection with a sharp increase toward the end of the symptomatic phase (11). The lifespan of a virus outside the cell is about 7.2 hrs (1-3).

A typical untreated disease course based upon CD4+ T-cell counts and viral level is simulated in Figure 1a,b (the initial period of viremia is not included in the model). The simulation in Figure 1a,b is in close agreement with a typical disease course (11). The initial virus level is determined by the model's parameters, which do not change throughout the course of the disease. This assumption is consistent with recent clinical findings that disease prognosis is correlated to a set-point of virus level established in each patient soon after the initial viremia, and viral levels and replication rates remain relatively stable after the set-point (5,8,15,16). In the model, different set-points are obtained by varying key parameter values.

In the model, treatment is incorporated as the reduction of two separate rates. The reduction of these rates provides treatment control variables corresponding to the intensity and velocity of drug action. The variables are the rate at which virus infects uninfected CD4+ T cells and the rate of virus influx into the plasma from the external lymphoreticular system. Reduction of this second rate is the most important for treatment outcome, since it is believed that as much as 98% of the virus in the circulating blood is contributed by the external lymphoid compartment (5,8,17). In the simulations, the dynamics in the lymphoid compartment are modeled as a viral source term rather than mechanistically, since limited data are available for this compartment (18). Models of combined plasma-lymph compartment dynamics will appear in future work. When treatment begins, the model assumes that a proportion of drug-sensitive virus mutates to drug-resistant virus. This proportion is also a treatment control variable corresponding to the combination of drugs used or the presence of genetic diversity at different disease stages (19).

The model distinguishes primarily between resistance and remission in the assumption of a threshold condition for the virus population in the plasma (and thus for the virus population in the lymphoid compartment). The threshold condition is incorporated into the rate that controls the contribution of drug-resistant virus from the external lymphoid compartment to the plasma. When treatment drives the plasma virus level below the threshold, the drug-resistant virus population does not emerge, and the drug-sensitive virus population falls to near 0. This threshold cannot be reached simply by gradually lowering the drug-sensitive virus population. Two additional factors must be considered: 1) when the virus population is above threshold, the high mutational capacity and short lifespan of the virus results in rapid production of drug-

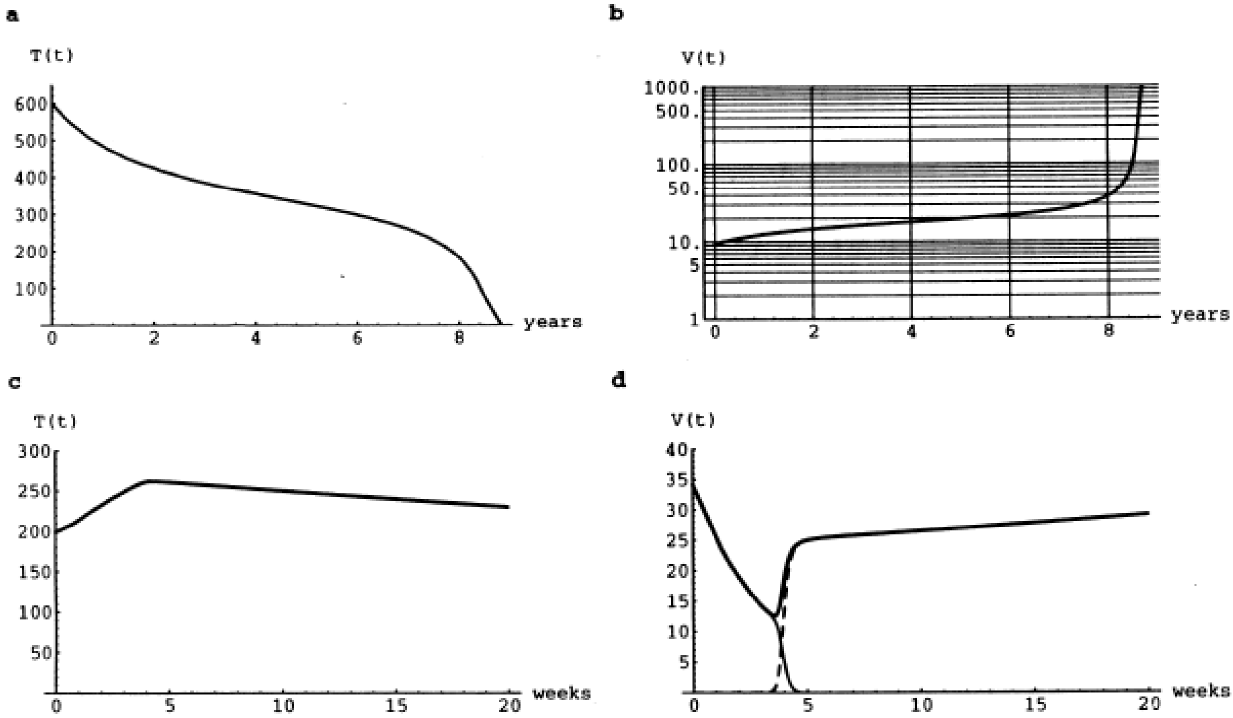

Figure 1a,b. A simulation of HIV dynamics for the model (A.1) - (A.3) with T(0) = 600/mm$^3$ and $V_s(0)$ = 10/mm$^3$. The curves correspond to data in (11). The set-point of the virus is in the middle range (15) and corresponds to a typical disease progression of about 9 years. The contribution to the plasma virus from the external lymphoid compartment is more than 90%, as may be computed from equation (A.3). The curves T(t) and $V_s(t)$ are approximately inversely proportional, as may be seen from equation (A.3) (the inverse proportionality is specific to a given set-point). In c,d, a simulation of a combined drug treatment corresponds to data in (9). The treatment begins with the uninfected CD4+ T-cell count at 200/mm$^3$, the infected CD4+ T-cell count at 9.5/mm$^3$, and the virus level at 34/mm$^3$ (these values are obtained from the simulation in a, b at 7.9 years). In d, the heavy line is the total virus population, the thin line is the wild-type virus population, and the dashed line is the drug-resistant virus population. Complete replacement of wild-type virus by resistant virus occurs by week 5. The treatment parameters are $c_1$=.5, $c_2$=.025, $c_3$=.15, and the resistance mutation parameter is q=$10^{-7}$.

resistant variants; and 2) as the virus population approaches the threshold, Darwinian competition gives competitive advantage to the resistant viral strain as the sensitive viral strain diminishes in fitness and in numbers.

To reach the threshold, the virus population must be brought down extremely fast before mutation and selection pressure allow resistant virus to propagate in the drug-altered environment. In the simulations, this rapid fall to the threshold can be achieved if treatment inhibits the rate of viral influx from the external lymphoid compartment sufficiently fast. The threshold value depends on the drugs used and the capacity of the virus to mutate against these drugs. In some patients, plasma levels were reduced by 99.9% or more, yet remission did not occur (1,2). In these cases, there may have been an extremely low threshold specific to the drugs used or a disproportionately lower suppression of virus in the lymphoid compartment than in the plasma.

### The Simulations

Computer simulations of treatment are given in Figure 1c,d and Figures 2-5. The initial values at the start of treatment for the simulations are obtained from the simulation in Figure 1a,b. In all the simulations, the parameters are the same, except for the parameters controlling treatment and the resistance mutation.

In Figure 1c,d, resistance is simulated. The simulation corresponds to composite data given (9) for patients receiving the drug combination saquinavir, zidovudine, and zalcitabine (the first is a protease inhibitor and the other two are reverse transcriptase inhibitors). Despite an impressive increase in the CD4+ T-cell counts in Figure 1c (significantly higher than typically seen with zidovudine alone [20-23]), the reduction of viral influx from the external lymphoid compartment is not fast enough or strong enough to bring the virus below the threshold for remission. The viral level thus rebounds after a few weeks, and the T-cell population resumes a decline.

In Figure 2, resistance is simulated corresponding to data for patients receiving therapy with strong reverse transcriptase inhibitors (10). In these simulations, the reduction of the viral influx from the external lymphoid compartment is higher than in the simulation in Figure 1d, which means that the effect of the drug is stronger and the decrease of the virus level is faster. The resistance mutation parameter is also assumed to be higher than in Figure 1c,d, which means that resistance develops sooner. The exponential rates of increase of CD4+ T-cell counts in Figure 2a are inversely correlated to CD4+ T-cell starting values, as are the times to the appearance of resistance. The exponential rates of viral decay (Figure 2b), as indicated by the slopes of their logarithmic plots, are approximately parallel and thus not correlated to

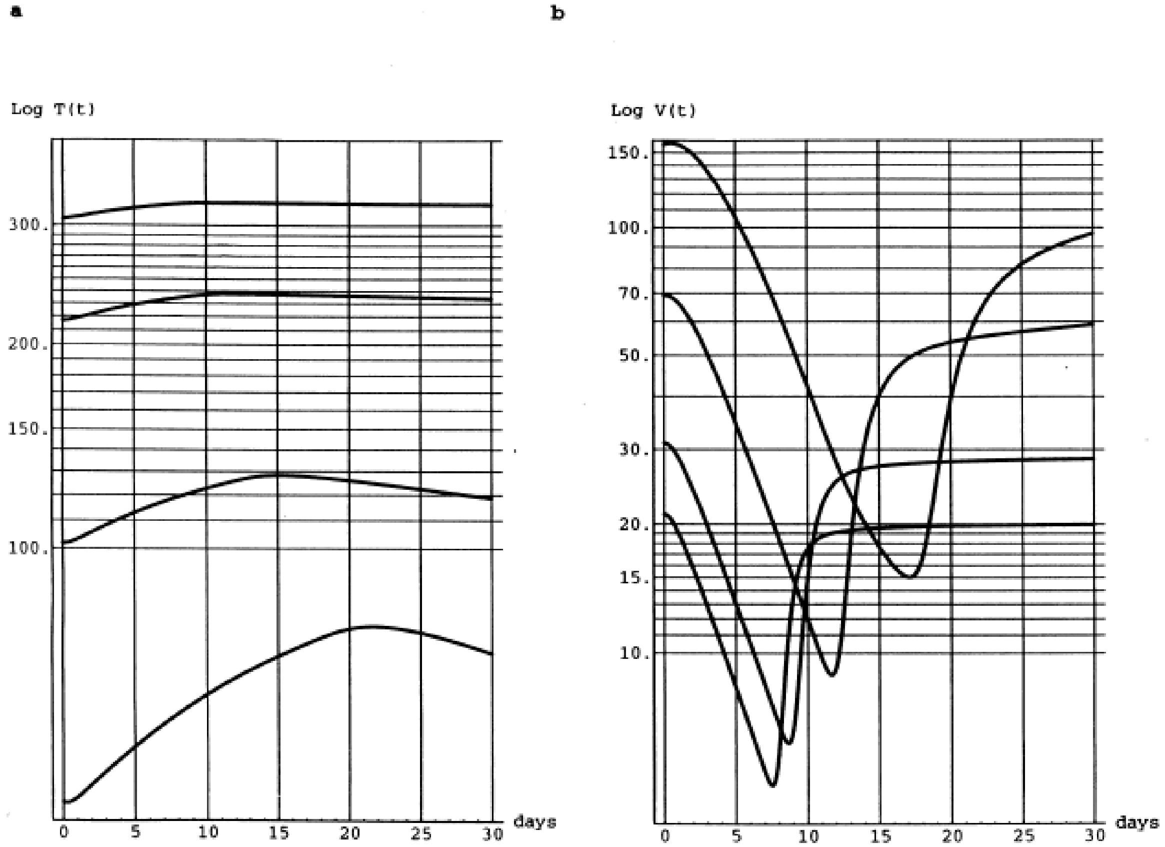

Figure 2. Four simulations corresponding to therapy (2). The simulations have a common viral set-point of disease progression with the treatment starting values T(0) = 306/mm$^3$ and $V_s(0)$ = 21/mm$^3$ (obtained from Figure 1a,b at 5.8 years), T(0) = 217/mm$^3$ and $V_s(0)$ = 31/mm$^3$ (obtained from Figure 1a,b at 7.7 years), T(0) = 100/mm$^3$ and $V_s(0)$ = 69/mm$^3$ (obtained from Figure 1a,b at 8.4 years), and T(0) = 43/mm$^3$ and $V_s(0)$ = 156/mm$^3$ (obtained from Figure 1a,b at 8.6 years). The rates of exponential increase in Figure 2a (approximately .03, .02, .01, .005) are inversely correlated to starting CD4+ T-cell counts, and the exponential rates of decay in Figure 2b (all about -.2) are not correlated to starting viral levels (different viral set-points would give different values for the parallel slopes) (1,2). The lack of correlation of viral decay rates is an indication of slower clearance of wild-type virus in the external lymphoid compartment. The time to the downward spike in Figure 2b is correlated to starting viral levels (1). The treatment parameters $c_1$=2.0, $c_2$=.17, $c_3$=.15 and the resistance mutation parameter q=$10^{-6}$ are the same in all four simulations.

treatment starting values. This lack of correlation has been reported in clinical data (1,2).

In the simulations in Figure 2, the lack of correlation can be explained by the failure of the treatment to suppress rapidly enough the viral production caused by the external lymphoid system. As this production is suppressed at faster and faster rates, the viral exponential decay rates approach the actual loss rates of the plasma virus, which in this model are correlated to the CD4+ T-cell levels. The decay rates (Figure 2b) do not yield the actual half-life of free virus, which is shorter. The difference is due to the incomplete inhibition of the external lymphoid viral production. The exponential rate of viral decay in patients undergoing treatment is claimed to correspond to the decay of noninfectious virus produced by CD4+ T cells infected after treatment begins by infectious virus present before treatment begins (where it is assumed that after treatment begins, all newly produced virions are noninfectious) (3). It is claimed that the reciprocal of the viral decay rate is the average lifespan of infected CD4+ T cells (3). The model considered here has a different interpretation of the effects of treatment, since the prouction of virus from the external lymphoid compartment is not immediately blocked by treatment and thus influences the viral decay rate.

In Figure 3, simulations are given with the treatment parameter corresponding to suppression of virus influx from the lymphoid compartment higher than in Figure 1c,d and the mutation parameter lower than in Figure 2. In Figure 3a, remission is achieved, but in Figure 3b,c,d, it is not (the threshold value is indicated by the horizontal lines). The concurrence of strong suppression of the lymphoid virus compartment, lower resistance mutation parameter,

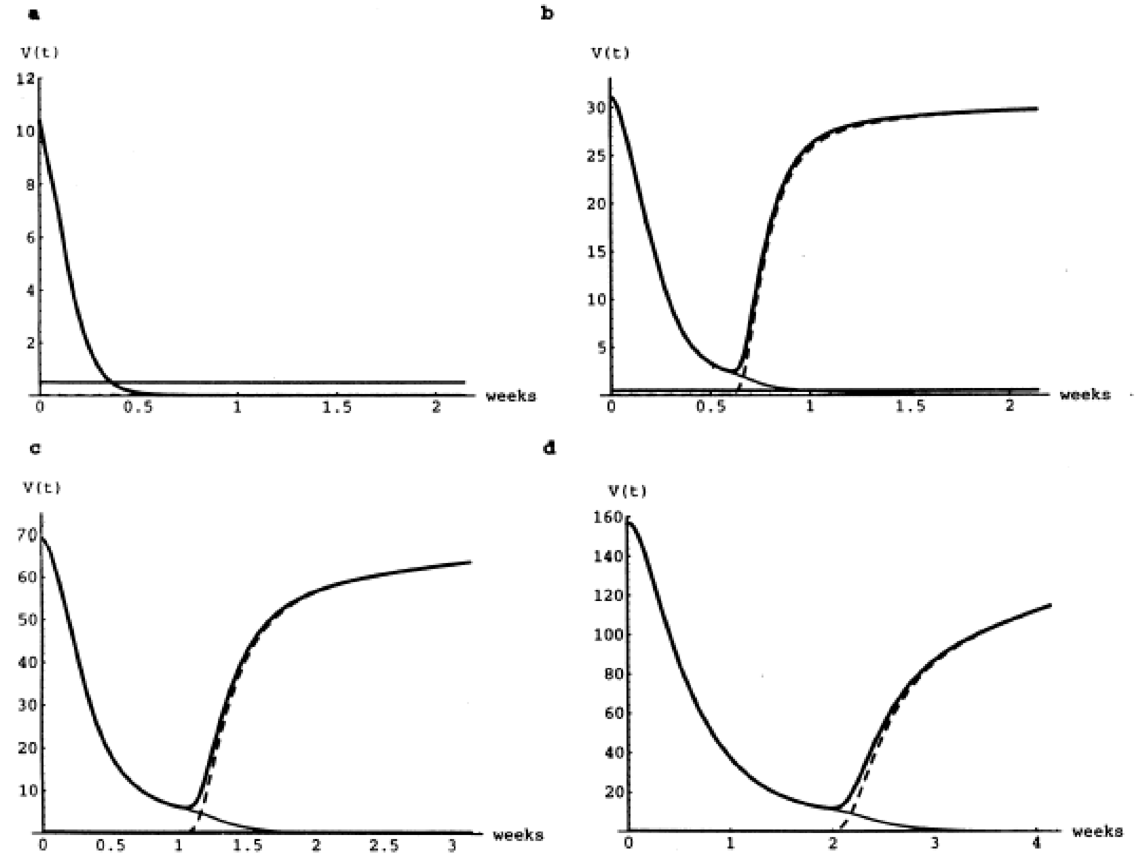

Figure 3. Treatment simulations for four starting viral levels. The simulations have a common viral set-point of disease progression, and the treatment starting values are from Figure 1a,b. For all four simulations, the treatment parameters are $c_1$=2.0, $c_2$=1.0, $c_3$=.1, the resistance mutation parameter is q=$10^{-7}$, and the threshold value is $V_0$=.5 (indicated by the horizontal line). The lowest starting viral level achieves remission (a), while the other three develop resistance.

and lower viral starting value allows the remission threshold to be reached (Figure 3a).

In Figure 4, treatment is simulated with an even higher value of the parameter corresponding to suppression of virus production from the external lymphoid compartment. Remission is achieved for the two lowest viral starting levels. As in Figure 2a, the exponential rates of increase of CD4+ T cells are inversely correlated to CD4+ T-cell starting values (an explanation in terms of the relative rates of changes in the differential equations for the CD4+ T-cell population is given in the Appendix). The exponential rates of viral decay (Figure 4b), however, are inversely correlated to increasing values of starting viral levels (in contrast with Figure 2b). When drug inhibition of virus in the lymph compartment is very high, the plasma viral clearance rate during treatment approaches the plasma viral clearance rate before treatment. In our models, it is assumed that the plasma viral clearance rate before treatment depends on CD4+ T-cell levels. The inverse correlation of plasma viral clearance rates during treatment to viral levels at the start of treatment is thus an indicator of higher viral clearance from the lymphoid compartment (an explanation in terms of the relative rates of change in the differential equation for the virus population is given in the Appendix).

In Figure 5a, treatment data for a patient receiving zidovudine, didanosine, and lamivudine are simulated (18; Figure 1d). This treatment induces a remission, even though the plasma virus does not fall below a nondetectable level. The plasma viral decay is approximately three times as fast as the lymph viral decay (18). This difference is incorporated into the treatment parameters for the simulation in Figure 5a. The two-phase plasma viral decay process (Figure 5a) matches the data (18) and is a strong indication that the rate of plasma viral decay is influenced by the slower rate of decay in the lymph system.

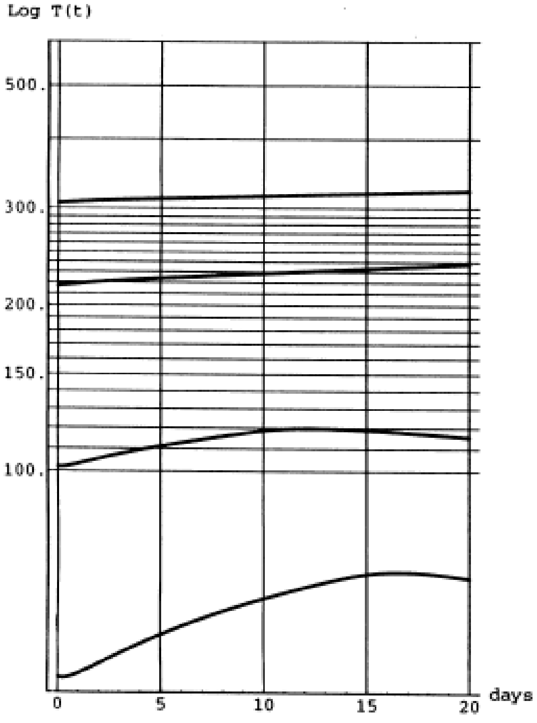

a b

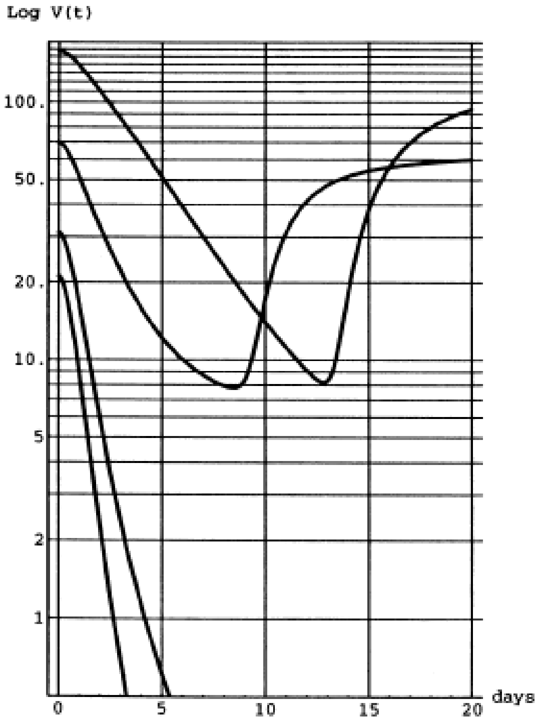

Figure 4. Four treatment simulations having a common viral set-point of disease progression. The treatment starting values are as in Figure 2. For all four simulations, the treatment parameters are $c_1$=2.0, $c_2$=2.0, $c_3$=.05, the resistance mutation parameter is q=$10^{-8}$, and the threshold value is $V_0$=2.0. Remission is achieved for the two lowest viral starting values, but the other two develop resistance. The viral exponential decay rates are -1.4, -.93, -.51, and -.26, which are inversely correlated to the viral starting values (an indication of rapid suppression of virus in the external lymphoid compartment).

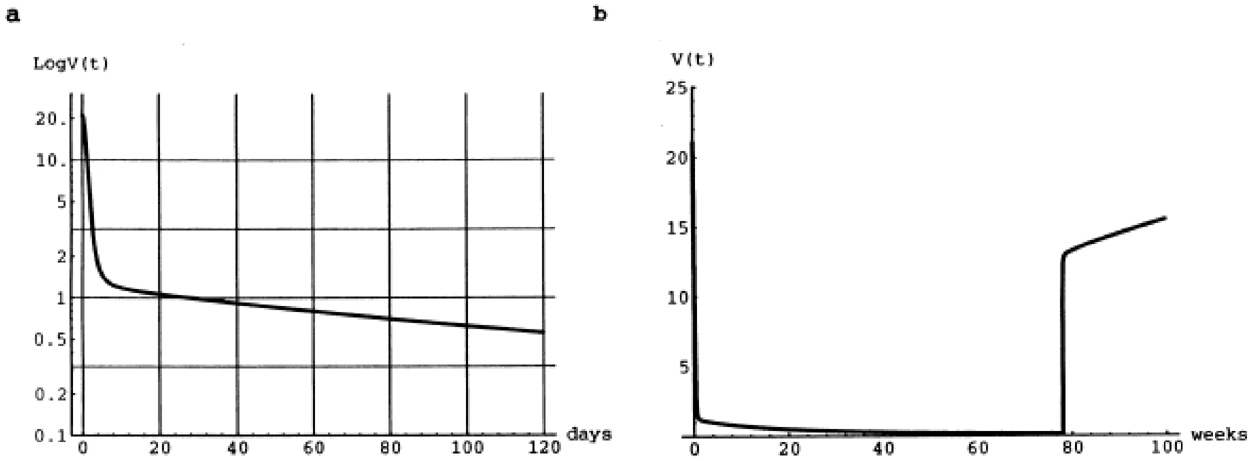

Figure 5. 5a simulates combined drug treatment data reported for a patient (18; Figure 1d). The treatment begins with the uninfected CD4+ T-cell count at 306/$mm^3$, the infected CD4+ T-cell count at 10/$mm^3$, and the virus level at 21/$mm^3$ (these values are obtained from the simulation in Figure 1a, b at 5.75 years). The treatment parameters are $c_1$=2.0, $c_2$=1.0, $c_3$=.15, the resistance threshold value is $V_0$=3.0, and the resistance mutation parameter is q=$10^{-7}$. Resistance does not develop, and the therapy results in remission. The plasma viral level shows a two-phase exponential decay, which is attributed to a slower drug-induced inhibition of virus in the lymphoid compartment. In 5b, the treatment simulation in 5a is continued for 78 weeks and then stopped. The virus population rebounds rapidly when treatment stops.

In Figure 5b, the treatment simulation (Figure 5a) is stopped at 78 weeks, whereupon the drug-sensitive virus population rebounds sharply (8). This simulation is consistent with the report of a patient undergoing combined therapy who sustained nondetectable levels of virus for 78 weeks and upon voluntarily stopping treatment experienced high levels of virus in the blood within 1 week (3). In this simulation the virus would rapidly become reestablished even after much longer treatment. The resurgence of the virus population in the simulation is due to the capacity of the virus to grow very quickly from extremely low levels, which is due to the incomplete drug-induced inhibition of the external lymphoid viral source (18). This incomplete inhibition can be attributed to the presence of latently infected CD4+ T cells in the lymphoid compartment. Resumption of treatment in the simulation could again induce a remission. The resurgence of virus when treatment is stopped (Figure 5b) would hold also in all the simulations of remission because the 0 viral level is unstable; and if the virus is not suppressed by drugs, it rapidly grows from even very low levels. This instability of the 0 viral level results from the large viral influx from the lymph system, which is required to produce the characteristic dynamics of HIV throughout its entire progression.

### Conclusions

Although computer models of HIV therapy are no substitute for clinical trials, they can bring into focus essential elements of the dynamic processes involved. The treatment simulations presented here identify the following qualitative dynamic elements involved in resistance and remission: 1) remission can occur if the viral production in the external lymphoid tissues is suppressed below a threshold level; 2) drug action must be strong enough and fast enough to drive the virus population to the threshold before resistant virus appears and propagates; 3) combination therapy or early treatment lowers the capacity of the virus to mutate to resistant strains and thus forestalls their emergence until the threshold is reached; and 4) stopping treatment even after an extended period of remission may result in a rapid rebound of the virus population.

The remission threshold is an abstract construct, and its quantitative value is relative to the capacity of the virus to mutate against a specific drug regimen. In the simulations, the threshold divides resistance and remission outcomes, and the dynamic developments in the first few days and weeks of drug administration are crucial in determining the outcome of therapy. Remission over extended time, however, may require continuing treatment to suppress low-level viral replication in the lymphoid tissues. The presence of even low-level viral production due to latently infected CD4+ T cells allows the possibility for the eventual evolution of drug-resistant viral strains.

### Appendix

For the model without treatment, it is assumed that only drug-sensitive virus, uninfected CD4+ T cells, and CD4+ T cells infected by drug-sensitive virus are present. The equations for the model without treatment are as follows (24):

(A.1): $dT(t)/dt = S(t) - \mu_T T(t) + p_1(t)T(t)V_s(t) - k_s V_s(t)T(t)$

(A.2): $dT_s(t)/dt = k_s V_s(t)T(t) - \mu_{Ti} T_s(t) - p_2(t)T_s(t)V_s(t)$

(A.3): $dV_s(t)/dt = p_3(t)T_s(t)V_s(t) - k_v T(t)V_s(t) + G_s(t)$

In (A.1) S(t) represents the external input of uninfected CD4+ T cells from the thymus, bone marrow, or other sources. It is assumed that there is a deterioration of this source as the viral level increases during the course of HIV infection. The form of this source is $S(t) = S_1 - S_2 V_s(t)/(B_s+V_s(t))$, where $B_s$ is a saturation constant (the various saturation constants in the model are designed to adjust the rate parameters to large changes in the population levels during disease progression or treatment). In (A.1) $\mu_T$ is the death rate of uninfected CD4+ T cells whose average lifespan is $1/\mu_T$ (25). In (A.1) the term $p_1(t)\, T(t)\, V_s(t)$ represents CD4+ T-cell proliferation in the plasma due to an immune response that incorporates both direct and indirect effects of antigen stimulation ($p_1(t) = p_1/(C+V_s(t))$, where C is a saturation constant). This term accounts for the above normal turnover of CD4+ T cells (other forms for this production have been used, including a logistic approach [26]). The form assumed here idealizes the growth mechanisms of CD4+ T cells, since subpopulations of antigen specific CD4+ T cells are not modeled. In (A.1) $k_s$ is the infection rate of CD4+ T cells by virus (it is assumed that the rate of infection is governed by the mass action term $k_s V_s(t)\, T(t)$). In the absence of virus the CD4+ T-cell population converges to a steady state of $S_1/\mu_T$.

In (A.2) there is a gain term $k_s V_s(t)\, T(t)$ of CD4+ T cells infected by drug-sensitive virus, a loss term $\mu_{Ti}\, T_s(t)$ due to the death of these cells independent of the virus population, and a loss term $p_2(t)\, T_s(t)\, V_s(t)$ dependent on the virus population due to bursting or other causes (where $p_2(t) = p_2/(C_i+V_s(t))$ and $C_i$ is a saturation constant). The dependence of the loss term $p_2(t)\, T_s(t)\, V_s(t)$ on $V_s(t)$ allows for an increased rate of bursting of infected cells as the immune system collapses and fewer of these cells are removed by CD8+ T cells.

In (A.3) the virus population is increased by the term $p_3(t)\, T_s(t)\, V_s(t)$, where $p_3(t) = p_3/(C_i+V_s(t))$. This term corresponds to the internal production of virus in the blood. The dependence of this term on $T_s(t)$ allows for a decreased rate of viral production in the plasma when the infected CD4+ T-cell population in the plasma collapses. Since most of the plasma virus is contributed by the external lymph source, the plasma virus population still increases steeply at the end stage of the disease. In (A.3) the virus population is decreased by the loss term $k_v\, T(t)\, V_s(t)$, which represents viral clearance. In (A.3) there is a source of virus from the external lymphoid compartment, which is represented by the term $G_s(t) = G_s V_s(t)/(B+V_s(t))$ (B is a saturation constant). This term accounts for most of the virus present in the blood (8).

The lifespans of infected CD4+ T cells and virus can be computed from the terms in (A.2) and (A.3) during the asymptomatic period of infection (when the rates of population increase are almost balanced by the rates of population decrease). The loss terms in (A.2) yield an average infected CD4+ T-cell lifespan of $1/\mu_{Ti} + p_2\, V_s(t)/(C_i + V_s(t))$, which decreases from $= 1/\mu_{Ti}$ to $1/(\mu_{Ti} + p_2)$ as $V_s(t)$ increases. The loss term in (A.3) yields an average virus lifespan of $1/(k_v T(t))$, which increases from $1/(k_v\, T(0))$ as T(t) decreases.

The equations for the model with treatment are as follows:

(A.4): $dT(t)/dt = S_0(t) - \mu_T T(t) + p_1(t)T(t)V(t) - (\eta_1(t)k_s V_s(t) + k_r V_r(t))\, T(t)$

(A.5): $dT_s(t)/dt = \eta_1(t)\, k_s V_s(t)\, T(t) - \mu_{Ti}\, T_s(t) - p_2(t)\, T_s(t)\, V(t)$

(A.6): $dT_r(t)/dt = k_r\, V_r(t)\, T(t) - \mu_{Ti}\, T_r(t) - p_2(t)T_r(t)\, V(t)$

(A.7): $dV_s(t)/dt = (1-q)p_3(t)T_s(t)V(t) - k_v T(t)V_s(t) + \eta_2(t)\, G_s V_s(t)/(B+V(t))$

(A.8): $dV_r(t)/dt = p_3(t)\, T_r(t)\, V(t) + q\, p_3(t)\, T_s(t)\, V(t) - k_v\, T(t)\, V_r(t) + G_r(V(t))V_r(t)/(B+V(t))$

In the model, treatment inhibits (with a delay) new infections of CD4+ T cells and inhibits (with a delay) the influx of virus from the external source. In equations (A.4) - (A.8) $V(t) = V_s(t) + V_r(t)$ is the total virus population at time t, and its inclusion in the rate coefficients results in competition between the sensitive and resistant viral strains. In these equations, treatment is modeled by the decreasing functions $\eta_1(t) = \exp(-c_1 t)$ (which inhibits the rate at which uninfected CD4+ T cells become infected) and $\eta_2(t) = \text{maximum}\{\exp(-c_2 t), c_3\}$ (which inhibits the influx of virus from the external lymphoid compartment). The parameters $c_1$, $c_2$, and $c_3$ control the speed and strength of the drug-induced inhibitions. The form of the treatment function $\eta_1(t)$ produces an eventual complete inhibition of infection of CD4+ T cells in the plasma but does not do so immediately upon treatment (1-3). The form of the treatment function $\eta_2(t)$ produces a delayed and incomplete suppression of viral influx from the external lymphoid system (18). Treatment does not affect the drug-resistant virus or the CD4+ T cells infected by drug-resistant virus.

When treatment begins, it is assumed that the source term of CD4+ T cells in equation (A.4) has the value $S_0(t) = \text{minimum}\{ S_0, S_1 - S_2V(t)/(B_s+V(t))\}$, where $S_0$ is the value of the source of CD4+ T cells when treatment is started ($S_0$ is obtained from the source function S(t) in the model without treatment). This assumption means that the source of CD4+ T cells does not increase once treatment begins but may decrease if the virus population later increases because of the development of resistance or the cessation of treatment.

In the model, it is assumed that there is no significant level of background resistant virus present to substantially affect the dynamics before treatment begins. After treatment begins, drug-resistant virus does become significant and is introduced into the virus population as a proportion q of the drug-sensitive virus population (19). It is not assumed that drug administration induces resistant mutations, but only that it gives selective advantage to them. The value of q corresponds to the capacity of resistant variants to mutate (larger q corresponds to monotherapy and smaller q to combined therapy). It is assumed that the external input of drug-resistant virus from the lymphoid compartment is controlled by the threshold function $G_r(V)$, where $G_r(V) = 0$ if V is less than the threshold value $V_0$ and $G_r(V) = G_s$ if V is greater than $V_0$. This assumption means that the capacity of the resistant virus to become established requires that the total virus population level remain above the threshold $V_0$.

The lack of correlation of the slopes in Figure 2b to starting CD4+ T-cell counts in Figure 2a can be explained in terms of equation (A.7). When treatment starts at time $t_0$, $G_s/(B+V(t_0)) \approx k_v T(t_0)$ (since the virus population is changing very slowly before treatment starts and the major source of virus present is due to the external source). After treatment starts, $dV_s(t)/dt \approx -\rho(t)V_s(t)$, where $\rho(t) = c_2(t)\, G_s/(B+V(t)) - k_v T(t)$. If $c_2(t) \approx 1$ (which corresponds to slow clearance of the external compartment), then $\rho(t) \approx 0$ and $\rho(t)$ does not have a strong dependence on $T(t_0)$ (as in Figure 2b). If $c_2(t) \approx 0$ (which corresponds to rapid clearance of the external lymphoid compartment), then $\rho(t) \approx k_v T(t)$ and thus shows a strong dependence on $T(t_0)$ (as in Figure 4b). A similar argument using equation (A.4) shows that when $c_1(t) \approx 0$ (as in Figures 2a and 4a), then the exponential rates of increase in CD4+ T-cell counts are inversely correlated to treatment CD4+ T-cell starting values.

The models described in this paper have evolved from earlier models by the authors (24,26-28). A major goal of the present work is to align the model simulations with an expanding base of data for HIV dynamics. The construction of the present models is based in part on theoretical assumptions about the rate changes of the interacting populations and in part on simulation of their known dynamic properties. Another major goal of the present work is to derive insight into the qualitative distinctions between monotherapy resistance and combined-therapy remission. In the model (A.4)-(A.8), this distinction resides in the mutation parameter q, which corresponds to the capacity of resistant virus to arise as a proportion of sensitive virus when the total virus population is above the threshold value $V_0$. When q is large (monotherapy), the total virus population does not fall below $V_0$, and resistant virus becomes established. When q is small (combined therapy) and the total virus population is brought below $V_0$ sufficiently fast in the first days and weeks of treatment, the resistant virus population cannot grow.

The models of this paper differ from earlier models (1-3). The models here describe disease progression, whereas others (1-3) describe short intervals of treatment from presumed dynamic steady states. The models here describe dynamics in the plasma, whereas others (1,3) describe dynamics in the total body. The models of this paper distinguish between the behavior of virus in the plasma and in the lymph system. In the models here, the virus increases steeply in the plasma but saturates in the lymph system. The assumption of a large saturating external source of virus to the plasma is required in this model for the simulation of data.

The models of this paper also assume that the viral clearance rate depends on the CD4+ T-cell level, whereas other models (1-3) assume that this rate is constant. This last assumption is required in our models to obtain the dynamics of disease progression. This assumption is reasonable in understanding how the virus population can increase steeply in the plasma as the CD4+ T-cell population in the plasma collapses. If the viral clearance rate in the plasma is independent of CD4+ T-cell levels, the steep increase of plasma virus (as much as 100-fold) at disease end would have to result from increased production. But the CD4+ T-cell population in the plasma collapses to near 0 so that this population cannot account for the high viral increase. In the models here, this steep increase of plasma virus results from the collapse of the immune response (which means that the plasma viral clearance rate should depend on CD4+ T-cell levels) and from a continuing influx of virus from the saturated external lymph source.

We provide a list of parameter values for the models with and without treatment (Table).

Table. Parameter values for the models

| Parameters and Constants | | | Values |
|---|---|---|---|
| $\mu_T$ | = | mortality rate of uninfected CD4+ T cells | 0.005/day |
| $\mu_{Ti}$ | = | mortality rate of infected CD4 + T cells | 0.25/day |
| $k_s$ | = | rate CD4+ T cells are infected by sensitive virus | 0.0005 $mm^3$/day |
| $k_r$ | = | rate CD4+ T cells are infected by resistant virus | 0.0005 $mm^3$/day |
| $k_v$ | = | rate of virus loss due to the immune response | 0.0062 $mm^3$/day |
| $p_1$ | = | production rate of uninfected CD4+ T cells | 0.025/day |
| $p_2$ | = | production rate of infected CD4+ T cells | 0.25/day |
| $p_3$ | = | production rate of virus in the blood | 0.8/day |
| $G_s$ | = | external lymphoid sensitive virus source constant | 41.2/$mm^3$ day |
| $G_r$ | = | external lymphoid resistant virus source constant | specified in text |
| $V_0$ | = | threshold value for remission | specified in figure legends |
| q | = | proportion of drug-resistant virus produced from wild-type virus | specified in figure legends |
| C | = | half saturation constant of uninfected CD4+ T cells | 47.0/$mm^3$ |
| Ci | = | half saturation constant of infected CD4+ T cells | 47.0/$mm^3$ |
| B | = | half saturation constant of external virus input | 2.0/$mm^3$ |
| $B_s$ | = | half saturation constant of CD4+ T-cell source | 13.8/$mm^3$ |
| $S_1$ | = | source of CD4+ T cells in absence of the disease | 4.0/$mm^3$ day |
| $S_2$ | = | reduction constant of CD4+ T-cell source | 2.8/$mm^3$ day |
| $c_1$ | = | treatment parameter for suppression of the rate of CD4+ T-cell infection by virus | specified in figure legends |
| $c_2$ | = | treatment parameter for suppression of the rate of virus contributed by the external lymphoid compartment | specified in figure legends |
| $c_3$ | = | treatment parameter for maximal suppression of virus contributed by the external lymphoid compartment | specified in figure legends |
| $\eta_1$ | = | treatment function for inhibition of the rate at which virus infects uninfected CD4+ T cells | specified in text |
| $\eta_2$ | = | treatment function for inhibition of the rate of virus influx from the external lymphoid system virus | specified in text |

### Acknowledgment

This work was supported under grant numbers DMS 9596073 and DMS 9631580 of the National Science Foundation.

Dr. Kirschner's research is in computational microbial pathogenesis in the Microbiology and Immunology Department, University of Michigan Medical School. Her work focuses on understanding the mechanisms for disease progression for such pathogens as HIV and *Helicobacter pylori*. She also studies the role of chemotherapy in disease dynamics as well as treatment strategies for these infections. Her recent work explores the role of the thymus in pediatric HIV infection.

Dr. Webb's research is in mathematical models of population dynamics; it includes theoretical investigations of differential equations that arise in models of biomathematics as well as computational investigations of specific models of biological populations, including models of the HIV-infected immune system, tumor growth, the spread of epidemics, and the blood production system.

### References

1. Ho DD, Neumann AU, Perelson AS, Chen W, Leonard JM, Markowitz M. Rapid turnover of plasma virions and CD4 lymphocytes in HIV-1 infection. Nature 1995;373:123-6.
2. Wei X, Ghosh SK, Taylor ME, Johnson VA, Emini EA, Deutsch P, et al. Viral dynamics in human immunodeficiency virus type 1 infection. Nature 1995,373:117-22.
3. Perelson AS, Neumann AU, Markowitz M, Leonard JM, Ho D. HIV-1 Dynamics in vivo: clearance rate, infected cell lifespan, and viral generation time. Science 1996;271:1582-6.
4. Piatak M, Saag MS, Yang LC, Clark SJ, Kappes JC, Luk KC, et al. High levels of HIV-1 in plasma during all stages of infection determined by competitive PCR. Science 1993;259:1749-54.
5. Richman DD. HIV therapeutics. Science 1996;272:1886-7.
6. Coffin JM. HIV population dynamics in vivo: implications for genetic variation, pathogenesis and therapy. Science, 1995;267:483-9.
7. Stephenson J. New anti-HIV drugs and treatment strategies buoy AIDS researchers. JAMA 1996;275:579-80.
8. Carpenter CJ, Fischl MA, Hammer SM, Hirsch MS, Jacobsen DM, Katzenstein DA, et al. Antiretroviral therapy for HIV infection. JAMA 1996;276:146-54.
9. Collier AC, Coombs RW, Schoenfeld DA, Bassett RL, Timpone J, Baruch A, et al. Treatment of human immunodeficiency virus infection with saquinavir, zidovudine, and zalcitabine. N Engl J Med 1996;334:1011-7.
10. Cohen J. Shooting for the moon with drugs. Science 1996;273:302.
11. Pennisi E, Cohen J. Eradicating HIV from a patient: not just a dream? Science 1996;272:1884.
12. Grody WW, Fligiel S, Naeim F. Thymus involution in the acquired immunodeficiency syndrome. Am J Clin Pathol 1985;84:85-95.
13. Philips AN, Sabin CA, Mocroft A, Janossy G. Antiviral therapy. Nature 1995;375:195.
14. Embretson J, Zupancic M, Ribas JL, Burke RA, Racz P, Tenner-Racz K, Haase AT. Massive covert infection of helper T lymphocytes and macrophages by HIV during the incubation period of AIDS. Nature 1993;362:359-62.
15. Ho D. Viral counts in HIV infection. Science 1996;272:1124-5.
16. Mellors JW, Rinaldo CR, Gupta P, White RM, Todd JA, Kingsely LA. Prognosis in HIV-1 infection predicted by the quantity of virus in plasma. Science 1996;272:1167-70.
17. Pantaleo G, Graziosi C, Demarest JF, Butini L, Montroni M, Fox CH, et al. HIV infection is active and progressive in lymphoid during the clinically latent stage of disease. Nature 1996;362:355-8.
18. Lafeuillade A, Poggi C, Profizi N, Tamalet C, Costes O. Human immunodeficiency virus type I kinetics in lymph nodes compared with plasma. J Infect Dis 1996;174:404-7.
19. McLean A, Nowak M. Competition between AZT sensitive and AZT resistant strains of HIV. AIDS 1992;6:71-9.
20. Fischl MA, Richmann DD, Hansen N, Collier AC, Carey JT, Para MF, et al. The safety and efficiency of AZT in the treatment of subjects with mildly symptomatic HIV type 1. Ann Intern Med 1990;112:727-37.
21. Graham NMH, Zeger SL, Park LP, Vermund SH, Detels R, Rinaldo CR, Phair JP. The effects on survival of early treatment of HIV infection. N Engl J Med 1992;326:1037-42.
22. Hamilton JD, Hartigan PM, Simberkoff MS, Day PL, Diamond GR, Dickinson GM, et al. A controlled trial of early vs late treatment with AZT in symptomatic HIV infection. N Engl J Med 1992;326:437-43.
23. Montaner JSG, Singer J, Schechter MT. Clinical correlates of in vitro HIV-1 resistance to zidovudine. Results of the Multicentre Canadian AZT trial. AIDS 1993;7:189-95.
24. Kirschner DE, Webb GF. A model for treatment strategy in the chemotherapy of AIDS. Bull Math Biol 1996;58:367-91.
25. Trough DF, Sprent J. Lifespan of lymphocytes. Immunol Res 1995;14:1-14.
26. Perelson AS, Kirschner DE, DeBoer R. The dynamics of HIV infection of CD4+ T cells. Math Biosci 1993,114:81-125.
27. Kirschner DE, Webb GF. Effects of drug resistance on monotherapy treatment of HIV infection. Bull Math Biol 1997.
28. Kirschner DE, Webb GF. A mathematical model of combined drug therapy of HIV infection. Journal of Theoretical Medicine 1997. In press.
